# Evidence for resource transfer via common endophyte networks

**DOI:** 10.1038/s41598-026-56653-9

**Published:** 2026-06-08

**Authors:** Philipp Waschk, Philipp Spiegel, Arthur Gessler, Matthias Saurer, Beatrice M. Bock, Wolfgang Hinterdobler, Mark A. Anthony

**Affiliations:** 1 Center for Microbiology and Environmental Systems Science, University of Vienna, Vienna, Austria; 2 Swiss Federal Institute for Forest, Snow, and Landscape Research (WSL), Birmensdorf, Switzerland; 3 Department of Environmental Systems Science, ETH Zürich, Zürich, Switzerland; 4 Department of Biological Sciences, Northern Arizona University, Flagstaff, AZ USA; 5 Center for Adaptable Western Landscapes, Northern Arizona University, Flagstaff, AZ USA; 6 MyPilz GmbH, Vienna, Austria

**Keywords:** Fungi, Trichoderma, Common mycorrhizal networks, Fusarium, Mucor, Common fungal networks, Ecology, Ecology, Microbiology, Plant sciences

## Abstract

Fungal symbionts play essential roles in ecosystems influencing plant development and biodiversity. Mycorrhizal fungi can form common mycorrhizal networks (CMNs) where a fungus connects the roots of at least two plants via continuous extraradical mycelium and transfers resources such as nitrogen and carbon. In addition to mycorrhizal fungi, there is another group of fungal mutualists known as endophytes. They also support plant development and may form common endophyte networks (CENs). Whether endophytes can transfer soil resources like nitrogen, carbon, and water through such networks remains an open question. To test this, we established a CEN experiment in split petri dishes involving *Arabidopsis thaliana* hosts and three phylogenetically diverse endophytes (*Trichoderma viride*, *Mucor hiemalis*, and *Fusarium temperatum*) to test whether resources like isotopically labelled amino acid ^15^nitrogen (N), amino acid ^13^carbon (C), ^15^N-ammonium, or deuterated water can be transferred by donor to receiver plants connected via CENs. We show that the tested endophytes can form CENs and transfer growth limiting resources from donor plant soil to receiver plant tissues. *F. temperatum* boosted plant growth by 38% relative to the uninoculated control, and it enriched plant ^15^N content derived from amino acids by 55%. Surprisingly, we also observed amino acid-derived ^13^C transport from donor plant soil to receiver plant tissues by *T. viride* (+ 2.83% > control). We also demonstrate that soil resource transfer, evaluated as isotope enrichment, by all three endophytes shifted in the presence of two versus a single host plant even when root systems were physically separated to avoid competition, underscoring that endophytic functioning, not just that of plants, also shifts when CENs are formed. Our results demonstrate that non-mycorrhizal fungi, like endophytes, can form networks similar to the idea of CMNs and transfer plant growth relevant resources. Endophytes display a broad array of symbiotic functions with their hosts, and formation of CENs may be a newly discovered component of their symbiotic tool kit.

**Supplementary Information:**

The online version contains supplementary material available at 10.1038/s41598-026-56653-9.

## Introduction

Cross-kingdom interactions with fungi strongly influence plant development and biodiversity^1^. Fungal symbionts are particularly important, with mycorrhizal fungi receiving the most attention because they form tight associations with plant roots and confer nutritional and pathogen resistance benefits^2–4^. Some mycorrhizal fungi form so-called ‘common mycorrhizal networks’ (CMNs) characterized by a mycorrhizal fungus connecting the roots of two or more plants via continuous extraradical mycelium^5,6^. CMNs can connect the roots of different plants^7^ and have been implicated in transporting water, nitrogen, and carbon^8–10^. While mycorrhizal fungi have received most attention for forming these cross-plant networks, there is another, separate group of plant-associated fungi known as endophytes. Endophytes may play similarly important roles in improving plant growth, increasing nutrient supply, and potentially even forming continuous mycelia networks to interconnect plants^11–16^. In this study, we define endophytes *sensu* Liao et al., (2025) as asymptomatic microbial partners that co-inhabit healthy internal plant tissues with the ability to confer benefits.

Endophytes can be found in nearly all living plants in natural ecosystems. Root inhabiting endophytes can improve host plant growth and nutrient uptake^17^. Despite their ubiquity and contributions to plant development, this group stands out as particularly understudied in ecological analyses, especially when colonizing non-grass hosts and on plant root systems where the capacity to form networks in the soil is possible^11,12,18–20^. Recently, evidence that endophytes can form CMN-like networks was first demonstrated. An endophytic *Alternaria* was shown to mediate transfer of a water-soluble dye injected into “donor plant” leaves into receiver plants^21^. This study establishes that “CENs” (common endophyte networks) can be formed among co-occurring grasses, and it raises the possibility that endophytes may more broadly engage in this type of mycelium establishment to transport soil resources that plants need for growth, such as nitrogen, water, and possibly even carbon^15^.

**Fig. 1:**
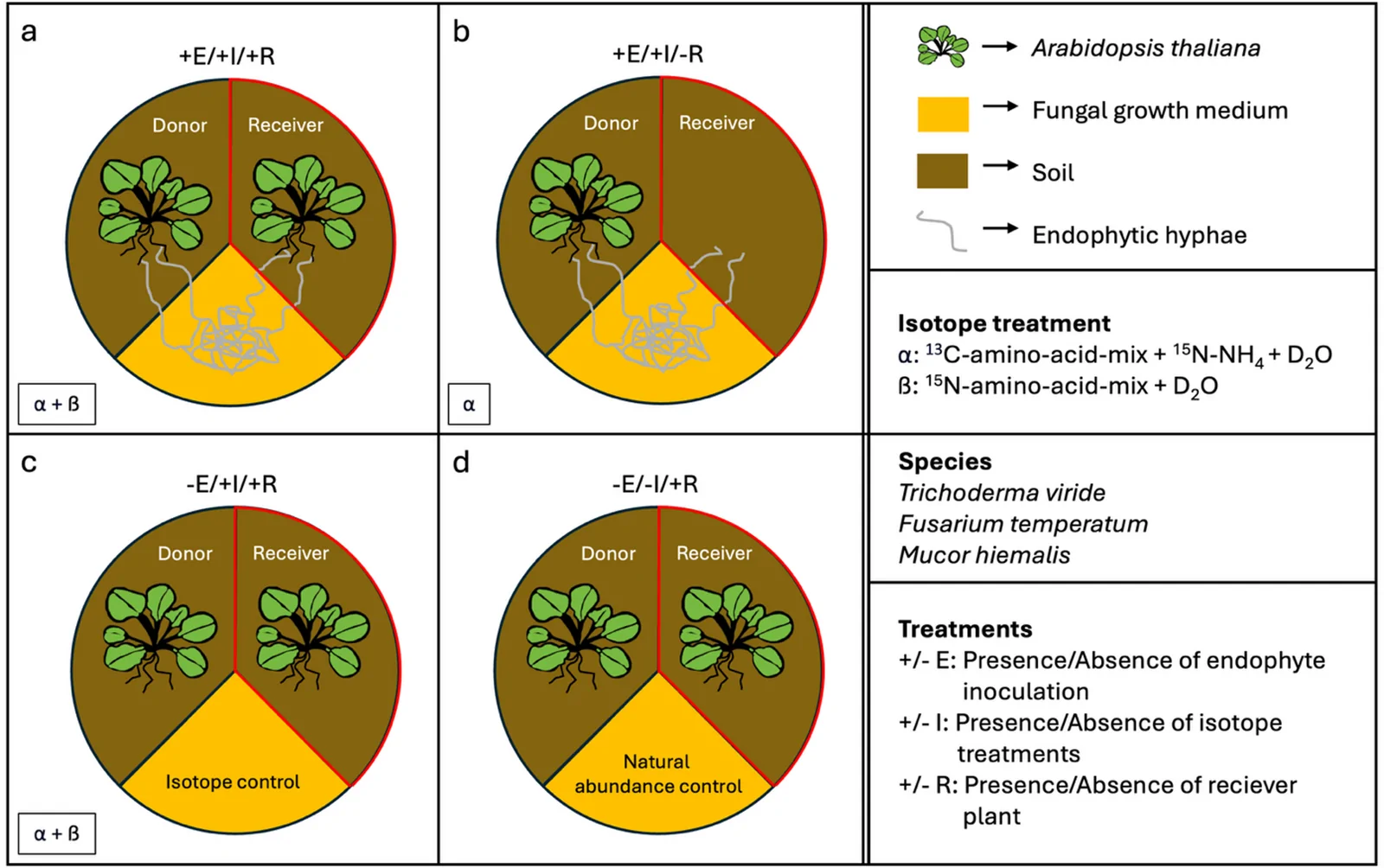
Experimental design to test for resource transfer, evaluated as isotope enrichment, via common endophyte networks. The experimental design consists of four distinct treatment levels aimed to quantify: resource transfer from donor compartment soils into receiver plants (**a**); resource transfer without a receiver plant present (**b**); non-fungal mediated resource transfer (**c**); and natural abundances of the isotope of the labelled substrate of interest (**d**) (see substrates tested in the legend). The receiver compartments used for the analyses are marked in red. See Supplementary Fig. 1 for pictures of inoculated plates.

To address this, we investigated whether CENs established by a range of endophytic species facilitate the exchange of nitrogen (ammonium, amino acids), carbon (amino acids), and water between plants colonized by the same fungal individual. Soil-derived water, nitrogen, and to a much lesser extent, photosynthetic-derived carbon are expected to be transported via CMNs (Simard et al., 2015), and thus we focused on soil resource versus photosynthetic carbon transfer. Like most studies tracking soil nutrient exchange via CMNs, this involves measuring enrichment of isotopically labelled substrates like ammonium from a “donor” compartment soil into a receiver plant with or without a contiguous fungal network. This should be conceptualized as comparing resource transfer from donor soil via a fungal network connecting both plants to a receiver plant. It is not necessarily directly from the donor plant to a fungal network connecting both plants to a receiver plant. We also included a treatment without a receiver plant which enabled us to test how fungal resource transfer, evaluated as isotope enrichment, from donor soil to receiver soil shifts in the presence versus absence of a CEN connecting two plants, allowing us to also test changes in fungal functioning (Fig. 1). Typically, the functioning of fungi is not the focus of CMN research where more attention is usually allocated to plant functioning^5^.

As a host plant, we used *Arabidopsis thaliana* and three different endophytic fungal species: *Fusarium temperatum*, *Mucor hiemalis*, and *Trichoderma viride*. We selected *A. thaliana* due to its fast growth and well described genome, because it is colonized by a large range of endophytic fungi, and because it is a model plant for non-mycorrhizal symbioses^18,22–27^. We selected the endophyte species because of their fast growth rates, broad phylogenetic range, and because of their ecological roles ranging from pathogenicity to mutualism. We hypothesize that all three endophytes facilitate resource transfer from the donor compartment to receiver side plant but that each species will provide different resources, with *T. viride* providing the most transfer given its well-described, positive effects on plant development^28^.

## Results

### Validation of the experimental system

To test whether factors apart from the inoculation with endophytes affected receiver plant physiological and isotopic signatures, we conducted tests to critically evaluate the experimental design. First, we confirmed that isotopically labelled nitrogen did not increase total plant nitrogen nor total soil nitrogen content on the receiver side (Suppl. Figure 3a-b), confirming that isotope additions did not have a fertilization effect on the receiver side. Growth of the donor and receiver plant on the same plate were not correlated and were thus considered independent (Suppl. Figure 3c). This is expected unless there were plate-level effects that coupled their growth because the plants were not uniform in size prior to out-planting into the plates. Donor and receiver plants also showed similarly high fungal root colonization (Suppl. Figure 5), demonstrating that both plants were simultaneously highly colonized. Additionally, we have found the type of colonization to be intracellular within root tissues for all three endophyte species (Suppl. Figure 6), showing that the contact of endophyte and plant was similar across all three endophyte species. Concurrent root colonization alongside visual fungal in-growth into both the donor and receiver side soils from the fungal compartment (Suppl. Figure 2) are signatures of hyphal continuity in the study system. For all subsequent analyses, we only looked at receiver plants to address receiver plant functioning and transfer.

There may be non-biological isotope transport via airborne substances or water flow that crossed the dividing barriers to the receiver plant. To test for this, we compared the isotope addition control without fungi (Fig. 1; -E/+I/+R) to the natural abundance control ((Fig. 1; -E/-I/+R; see methods). There were no differences between the natural abundance and isotope controls for ^15^N and ^13^C for any of the measured parameters, confirming that there was no alternative transport mechanism for carbon and nitrogen onto the receiver side in our experimental set-up (Suppl. Figure 4a-b). However, for the added D_2_O, we observed elevated deuterium levels in the receiver plants of the isotope controls compared to the natural abundance control (*p* = 0.0007, Suppl. Table 1). Surprisingly, this same pattern was not observed in the receiver soil. Therefore, an alternative transport of deuterated water to the plant but not the soil, like transport by evaporation and subsequent condensation, was present in our experimental set-up (Suppl. Figure 4c). Hereafter, only the natural abundance control was used to compare to the inoculation treatment levels (hereafter referred to as “control”) unless stated otherwise (e.g., in the D_2_O plant treatment where we also included the isotope control due to the effect mentioned above).

**Fig. 2:**
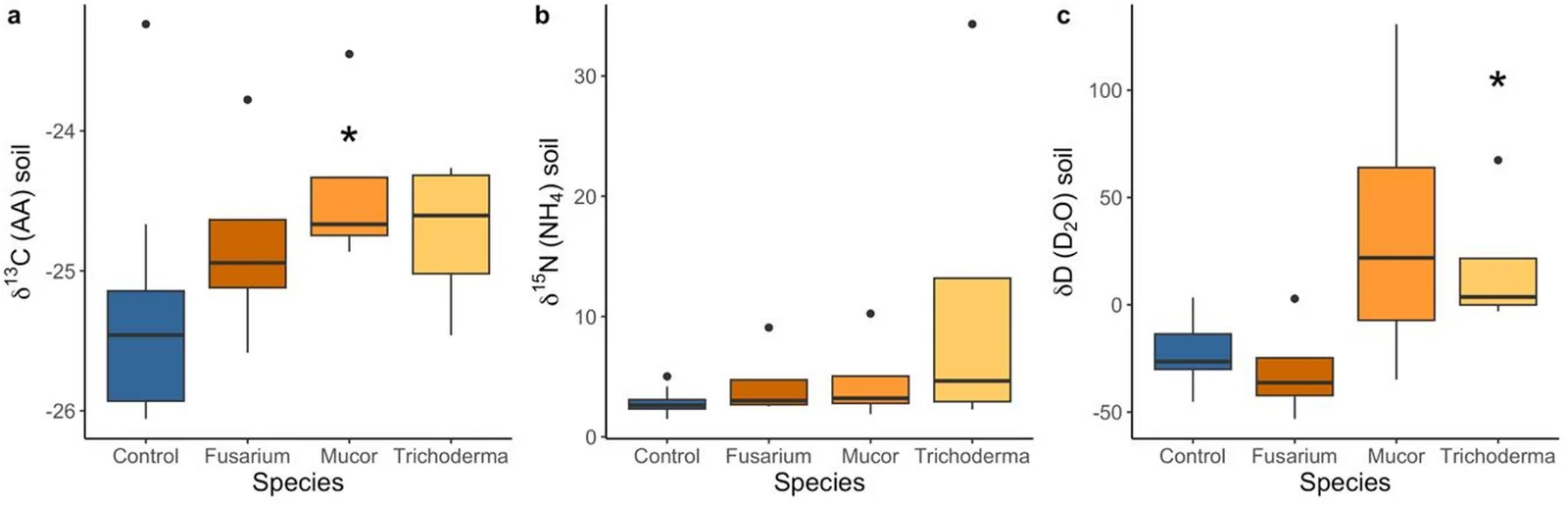
Isotopic analyses of soil without a receiver plant. Amino acid-derived δ^13^C in soil showing significant increase of δ^13^C in the *M. hiemalis* treatment (*p* = 0.05) (**a**). NH_4_^+^-derived δ^15^N in soil (**b**). D2O-derived δD in the soil showing significant increase of δD by *T. viride* (*p* = 0.04) (**c**). Note that a ‘*’ means *p* ≤ 0.05. For visualization purposes, the natural abundance control with receiver plant has been added to the figure.

Finally, we included plates with no receiver plants (Fig. 1; +E/+I/-R) and analyzed their receiver soil for isotopic signatures to account for the possibility that the endophytes transported isotopes to the receiver compartment independent of a receiver plant being present, and thus to account for the deposit of isotope-containing compounds through fungal necromass and exudates. We only tested this using ^13^C-labelled amino acids, ^15^N-labelled ammonium and D_2_O. When the added isotopic compound on the donor side was ^13^C-labelled amino acids, inoculation with *M. hiemalis* increased ^13^C levels of the receiver soils compared to the controls with no fungus but a receiver plant suggesting immobilization of carbon from the donor side into fungal biomass on the receiver side (*p* = 0.05, Fig. 2a). ^15^N levels were not significantly different to the control without an inoculated fungus but with a receiver plant present when the added isotopic compound on the donor side was ^15^N-labelled ammonium (Fig. 2b). There was a significant effect of the fungal inoculations on deuterium levels of the receiver soils (*p* = 0.049, Suppl. Table 2). Inoculation with *T. viride* (*p* = 0.04) increased deuterium levels of the receiver soils of the plates without a receiver plant compared to controls without fungi but including a receiver plant suggesting immobilization of water or deuterium-containing compounds from the donor side into fungal biomass on the receiver side (Fig. 2c). Inoculations with *F. temperatum* and *M. hiemalis* did not show any significant changes in deuterium signatures.

### Effects of endophyte inoculation on plant growth and nutrient content

**Fig. 3:**
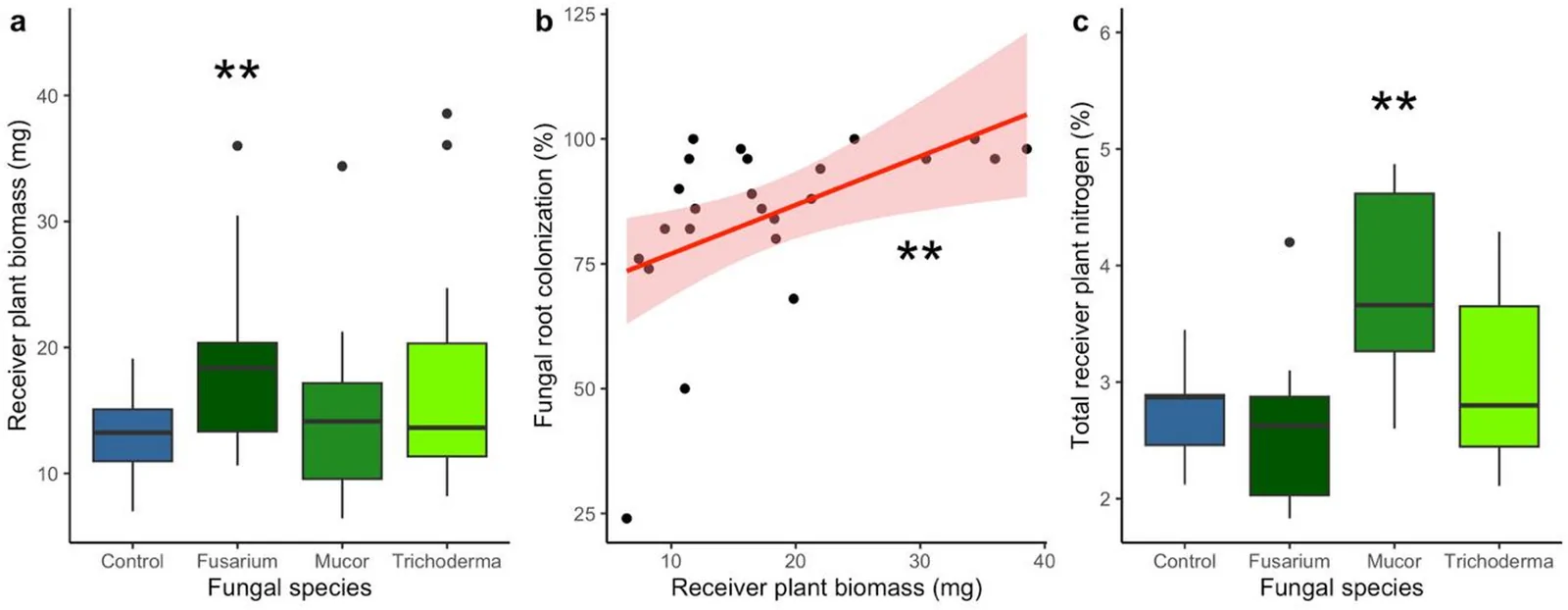
Physiological analyses of the receiver plants. Variation in plant biomass growth showing a significant increase in plant biomass by *F. temperatum* (*p* = 0.01) (**a**). Linear correlation of fungal root colonization and plant biomass with control treatments excluded (*p* = 0.01, *r* = 0.51) (**b**). Changes in total plant nitrogen content by fungal species showing significant increase of total plant nitrogen by *M. hiemalis* (*p* = 0.001) (**c**). Note that ‘**’ means *p* ≤ 0.01.

Physiological analyses of receiver plants were conducted to assess whether inoculation with endophytes affected plant growth and health. Inoculation with *F. temperatum* increased plant biomass in this experimental setup (*p* = 0.01) and inoculation with *T. viride* marginally increased plant biomass (*p* = 0.07, Fig. 3a). Further, extent and type of fungal root colonization of the receiver plants did not vary across the three endophyte inoculation treatments, meaning that differences in isotopic signatures across fungal species cannot be tracked back to different root colonization characteristics but rather relate to intrinsic differences across fungal species (Suppl. Figure 5,6). Further, fungal root colonization of the receiver plants was positively correlated with plant growth across all endophyte species (*p* = 0.01, *r* = 0.51, Fig. 3b). This suggests that a higher degree of root colonization by fungi boosts plant growth, an expected outcome for mutualistic, endophytic fungi, though it is also important to acknowledge that endophyte colonization is also regulated by host plants^29^. Root colonization of receiver plants was not correlated with plant growth in the control treatments (-E) suggesting that plant growth stimulation was conferred by the inoculated endophytes and not by fungi that were potentially carried over from the unsterile soil from before outplanting (Suppl. Figure 3 d). The potential carryover of exogenous fungi prevents attributing observed effects solely to the inoculated fungi because interactions with exogenous fungi may have influenced fungal functioning. Sequencing inoculated root systems to confirm the identity of established fungi would strengthen interpretation. Nevertheless, there was a significant effect of the fungal inoculations on the total plant nitrogen content of the receiver plants (*p* = 0.001). Inoculation with *M. hiemalis*, but not with *T. viride* nor *F. temperatum*, increased plant nitrogen content in the receiver plants compared to the controls (*p* = 0.001, Fig. 3c).

### Evidence for resource transfer from donor to receiver via endophytes

**Fig. 4:**
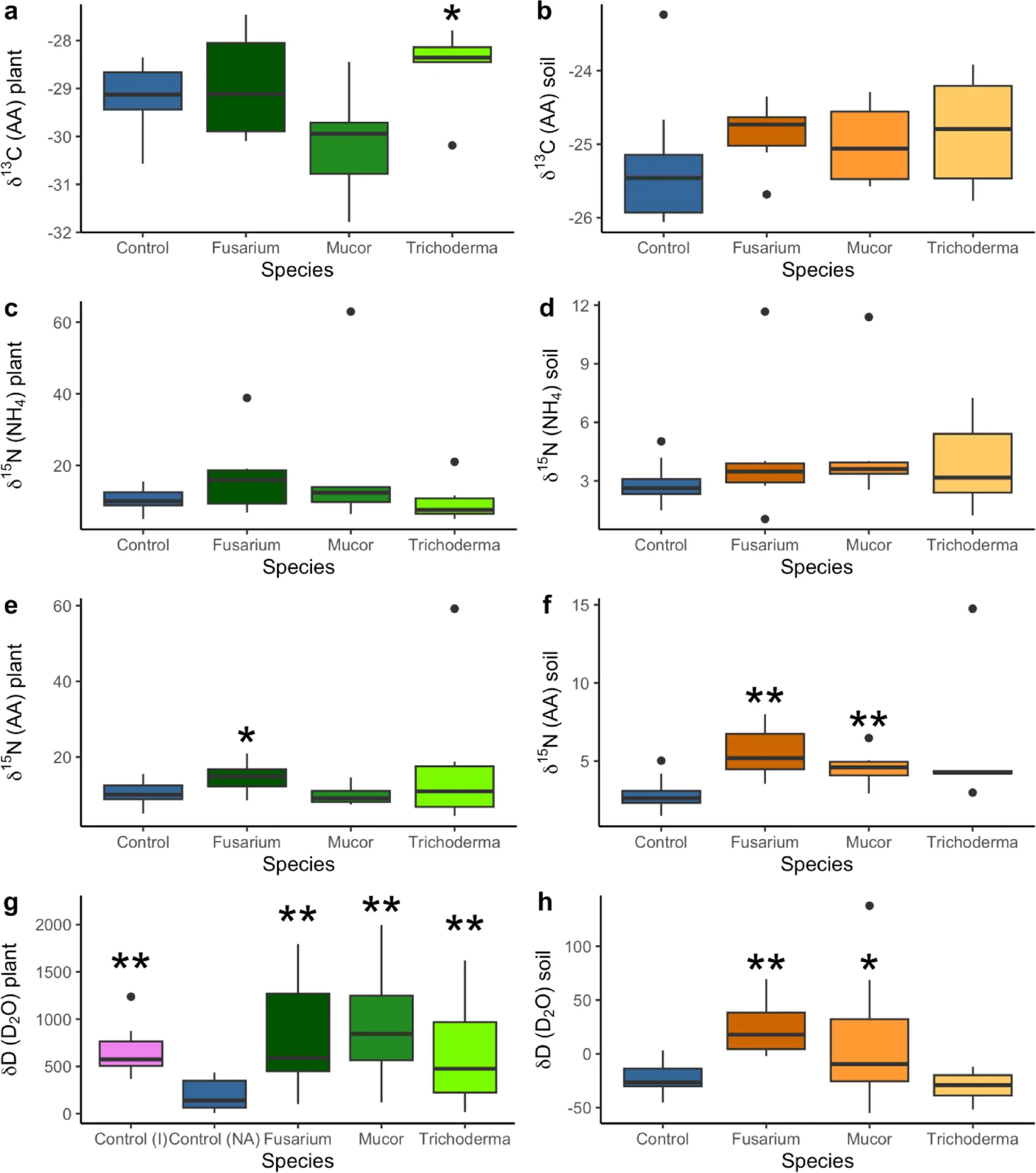
Isotopic analyses of receiver plants under fungal inoculation treatments. Amino acid-derived δ^13^C in the plant (**a**) and soil (**b**) showing significant increase of δ^13^C in plants by *T. viride* (*p* = 0.049) (**a**). NH_4_^+^-derived δ^15^N in the plant (**c**) and soil (**d**). Amino acid-derived δ^15^N in the plant (**e**) and soil (**f**) showing significant increase of δ^15^N by *F. temperatum* in plants (*p* = 0.017) and soil (*p* = 0.001) and by *M. hiemalis* in soil (*p* = 0.007). δD in the plant (**g**) showing significant differences between the natural abundance control (Control (NA)) and the isotope control (Control (I); *p* = 0.0007, Suppl. Figure 4c), *F. temperatum* (*p* = 0.002), *T. viride (p* = 0.0019) and *M. hiemalis* (*p* = 0.01) were all higher than the natural abundance control (Control (NA)) but not different from the isotope control (Control (I)). D2O-derived δD in the soil showing significant increase of δD by *F. temperatum* (*p* < 0.0001) and *M. hiemalis* in soil (*p* = 0.04) (**h**). Note that ‘*’ means *p* ≤ 0.05; ** means *p* ≤ 0.01).

When ^13^C-labelled amino acids were added to the donor side, there was a significant effect of the fungal inoculations on ^13^C-levels of receiver plants (*p* = 0.05). Inoculation with *T. viride* increased receiver plant ^13^C derived from labelled amino acids (*p* = 0.049), but not in the receiver soils compared to the control (Fig. 4a-b). Importantly, inoculation with *T. viride* also did not increase ^13^C-levels in receiver soils in the absence of a receiver plant compared to controls with a receiver plant (Fig. 2a). This suggests that *T. viride* did not simply transport ^13^C from amino acids into donor soil but rather facilitated direct carbon transport from the donor side to the receiver plant and that the transfer was contingent upon the presence of a receiver plant. In contrast, neither inoculation with *M. hiemalis* nor with *F. temperatum* had an effect on the^13^C levels of receiver plants nor soils compared to the control (Fig. 4a, b).

When ^15^N-labelled amino acids were added to the donor side, there was a significant effect of the fungal inoculations on ^15^N-levels of receiver soils (*p* = 0.01). Inoculation with *F. temperatum* also increased ^15^N levels of receiver plants by 55% compared to the control (*p* = 0.017; Fig. 4e). *F. temperatum* also elevated ^15^N levels in soil (*p* = 0.001; Fig. 4f). As a result, we cannot elucidate whether observed ^15^N in plants was provided indirectly via root uptake from fungal exudated ^15^N bearing compounds into receiver plant soil and/or potentially directly via continuous hyphal transport from the donor side. Inoculation with *M. hiemalis* increased ^15^N levels in receiver soils (*p* = 0.007) but not plants (Fig. 4e, f). *M. hiemalis* also transferred less ^15^N than *F. temperatum.* Inoculation with *T. viride* did not significantly change ^15^N-levels in plant or soil. We did not assess amino acid-derived ^15^N transfer to receiver soils in the absence of a receiver plant to test for fungal-mediated N transfer independent of a receiver plant (see *Study limitations* section) but based on the ^13^C-labelled amino acids and ^15^N-ammonium treatment (see above), we infer that transfer of amino acid-derived ^15^N to the receiver soil also required a receiver plant, providing support to the idea that amino acid-derived resource transfer to receiver side, evaluated as isotope enrichment, occurs via continuous fungal networks between two plants. When ^15^N-labelled ammonium was added to the donor side, we did not find ^15^N transfer to the receiver soil or plant for any endophyte species.

We originally assumed that the short time frame between D_2_O addition and sampling would not allow for significant exchange of deuterium from D_2_O with hydrogen from organic matter and transport of these D-enriched compounds, so that the detected changes in δD represent actual relocation of D_2_O. When D_2_O was added to the donor side, there was a significant effect of the fungal inoculations on the deuterium levels of the receiver plants (*p* = 0.02) and receiver soils (*p* = 0.0001). Inoculation with *F. temperatum* (*p* < 0.0001) and *M. hiemalis* (*p* = 0.04) increased deuterium levels of receiver soils compared to the control (Fig. 4h). Further, inoculations with *F. temperatum* and *M. hiemalis* did not affect deuterium levels in receiver soils in the absence of a receiver plant compared to controls with a receiver plant (Fig. 2c). This suggests a receiver plant-dependent water transport from donor to receiver soil via *F. temperatum* and *M. hiemalis.* Like the results of ^15^N from amino acids, *M. hiemalis* transferred less deuterium than *F. temperatum. T. viride* only transferred water to the receiver soil in the absence of a receiver plant (*p* = 0.04, Fig. 2c). The isotope control of deuterium in plants was significantly higher than the corresponding natural abundance control (*p* = 0.0007, Fig. 4g, Suppl. Figure 4c). Compared to the natural abundance control, all fungal treatments showed different plant deuterium levels (*p* < 0.05). Compared to the isotope control, none of the fungal treatments showed different plant deuterium levels, suggesting that an alternative, non-fungal pathway like evaporation and subsequent condensation may also explain significant deuterium enrichment in plants inoculated with different fungi (Fig. 4g).

## Discussion

Transport of resources via endophytic fungal networks extends the established role of non-mycorrhizal fungi beyond affecting a single host plant towards simultaneously impacting interconnected plants. In this study, we observed transfer of amino acid-derived resources, evaluated as isotope enrichment, from donor plant soils into receiver plant tissues by endophytes. Further, we found different resource allocation patterns among the tested species, and that fungal functioning shifted when engaged in a CEN versus symbiosis with a singular plant. This suggests that endophytes may play a previously unknown role in transferring soil resources via CENs, and that endophyte identity influences which types and magnitude of resources get transferred.

### Evidence that the tested fungal species are mutualistic endophytes

All three fungal species had beneficial effects on host plants in our experimental system. Across all species, root colonization was positively correlated with plant growth, which would be expected if fungi and plants acted mutualistically. Though not affecting total plant biomass, *M. hiemalis* increased total plant nitrogen. It did not transfer any of the labelled compounds potentially directly to the plant. It may therefore benefit plants by stimulating decomposition under receiver plants without forming CENs. *T. viride* also stimulated plant growth by 12%, but this effect was only marginally significant. *F. temperatum* significantly enhanced plant growth by 38% relative to the control. This result stands in contrast to it being previously described as a hemibiotrophic pathogen^30–32^, but it is consistent with other research showing that *Fusarium* species can more broadly act endophytically by stimulating plant growth hormone production and resource uptake^26,28,33^. In this study we show that *F. temperatum* can act mutualistically during this plant ontogenetic stage, and that all three tested fungi confer positive impacts on plant development.

### Evidence for amino acid nitrogen and carbon transfer by CENs

We identified endophyte-mediated transfer of amino acid nitrogen and carbon from donor soil compartments into receiver plants. Importantly, both receiver and donor plants were concurrently colonized by the same fungal genet. This was confirmed visually by the growing colony from the inoculation compartment extending into both soil compartments and at a finer scale using microscopy to confirm root colonization. This strongly suggests that there was hyphal continuity between the donor and the receiver plants in this study system, and that resource transfer, evaluated as isotope enrichment, occurred via a common endophyte network.

*F. temperatum* transferred amino acid nitrogen from the donor compartment to receiver plants. ^15^N enrichment in receiver plant tissues may have been derived from direct plant uptake of ^15^N bearing compounds in the soil since soil ^15^N levels were also elevated by inoculation. This ^15^N would have come from fungal exudates or necromass. This may also suggest direct transfer via hyphal continuity from the donor to receiver side. While these two possibilities cannot be teased apart in our study, it is likely the ^15^N in the receiver compartment soil is immobilized in fungal biomass since the study time frame was shorter than typical soil fungal turnover rates^34^. This would suggest that the transfer was direct from fungus to plant. This interpretation is also supported by our results of other fungi that did not enrich plant ^15^N levels. Inoculation with *M. hiemalis* elevated receiver soil ^15^N but without detectable enrichment in receiver plants, suggesting that observed ^15^N in the soil was immobilized in fungal tissues otherwise it would be expected to be readily taken up by the plant. Regardless of the mechanism, *F. temperatum* elevated plant ^15^N level when connected to more than one plant, and it did not translocate ^15^N from amino acids nor ammonium into receiver compartment soils in the absence of a receiver plant, establishing the importance of having more than one interconnected plant for resource transfer.

We also tracked *T. viride* transferred amino acid carbon from the donor compartment into receiver plants. Importantly, ^13^C levels in the receiver soil were comparable to natural abundance levels, which suggests a direct transfer of carbon from the donor compartment into the receiver plant via a *Trichoderma* CEN. It is important to note that ^13^C transfer from amino acids, while significantly higher than the control, was low (+ 2.83% higher than the natural abundance control), and thus, the ecological importance of CEN-based C transfer from amino acids may be minimal, similar to photosynthetic carbon transport via CMNs^22^. However, based on previous studies, and our own observations of enhanced development, *T. viride* promotes the growth of *A. thaliana* via elevated H+-ATPase activity^35^, and even without direct contact via plant-growth promoting volatile organic compounds^36^. Our findings may be an additional mechanism among the many by which *Trichoderma* stimulates plant growth.

*F. temperatum* and *M. hiemalis* also translocated D_2_O (and simultaneously ^15^N from amino acids) from the donor side into the receiver side soils. This transfer was also contingent upon the presence of a receiver plant and was not observed when receivers were absent. We did not find ammonium-derived nitrogen transfer suggesting the preference of organic nitrogen sources by these endophytes. While plant D was also elevated across all fungal treatments relative to the natural abundance control, they were not higher than the isotope control, suggesting non-fungal mediated D_2_O uptake in the plants. We know from an earlier study demonstrating that endophytic *Alternaria alternata* can transfer a visible dye serving as a tracer for water when injected into donor plant leaves into receiver plant tissues^21^. *A. alternata* is also a root endophyte, and thus, there may also be fungal-mediated water transfer into plant tissue in our study system but not at levels that can be distinguished from non-fungal pathways.

Resource transfer by *M. hiemalis*, evaluated as isotope enrichment, differed in the presence or absence of a receiver plant. We observed a switch of translocation of ^13^C from amino acids to the receiver side soil in the absence of a receiver plant to the transfer of D_2_O and ^15^N from amino acids in the presence of a receiver plant. While disproportionate focus in CMN research is typically on plant growth and resource uptake^5^, this result strongly suggests that endophytic fungi themselves functionally respond to the presence of more than one host plant, with implications for CEN resource exchange. We suggest that *M. hiemalis* is more dependent on soil carbon compounds from the donor side in the absence of a receiver plant, but when simultaneously connected to both donor and receiver plants, hosts may supply the fungus with plant carbon and the fungus can switch to acquiring more soil-derived nitrogen and water. This type of trophic flexibility may be largely unique to CENs since a large diversity of endophytes possess saprotrophic capacities while mycorrhizal fungi have mostly lost this ability^3,18,37–42^. Future research should balance how changes in both host plants and fungal functioning shift when endophytes produce continuous fungal networks because their distinct trophic flexibility differentiates them from CMNs.

### Common fungal network resource niche partitioning

Resource type and transfer rate varied among the three studied endophytes. *F. temperatum*, and possibly also *M. hiemalis* provided nitrogen and increased water transport while *T. viride* provided carbon. *Fusarium* also translocated more nitrogen and water than *Mucor*. To capture the idea that different endophytes contribute unique benefits to hosts when forming CFNs consisting of multiple CENs (or other CFNs), we propose the concept of ‘common fungal network resource niche partitioning (CFN-RNP)’. Since many endophytes co-exist in host plant roots in natural environments^24^, each endophyte could contribute individual benefits to a CEN when simultaneously colonizing multiple host plants, underscoring the potential importance of endophyte biodiversity. Previous research has demonstrated that soil endophyte biodiversity is positively linked to plant growth^12^, and while the involvement of CENs in natural systems remains untested, CFN-RNP provides an important framework for incorporating ecological concepts into CFN research. We suggest that further studies test the effect and transfer outcomes of resources in inoculations with multiple endophytic species to account for more complex network dynamics.

### Study limitations

This study is the first to show that soil resources (C and N) can move from donor soils to receiver plants when both are colonized by a single endophytic fungal genet. Although future field tests are needed, several limitations of our controlled experiment complicate interpretation. Because we lacked full isotope controls for the + E/+I/−R treatment (e.g., −E/−I/−R, −E/+I/−R) and did not include a treatment without a donor plant, we cannot disentangle all pairwise plant-fungal functional isotope changes. We designed the experiment to use ¹³C-labelled amino acids as a proxy to infer non-fungal transfer of amino acid-derived resources; however, because C and N moved independently in our study system, ¹³C data cannot be used to estimate ¹⁵N dynamics. As a result, we cannot disentangle amino acid-derived ¹⁵N transfer in the absence of fungi. We also cannot compare the magnitude of fungal transfer of the studied resources, evaluated as isotope enrichment, to the receiver side in the presence versus absence of a donor plant. This is a major limitation of the experimental design. However, both donor and receiver plants were highly colonized, and the presence of two plants (with or without receiver plants) altered transfer patterns. This indicates that removing the donor plant would also alter resource movement, given that plants were experimentally equivalent aside from isotope addition, and the fungus does not distinguish between donor and receiver plants. Finally, plant tissue controls for D_2_O were elevated above natural abundance and similar to fungal treatments, and despite minimizing the timeframe between D_2_O addition and sampling, this effect remained substantial suggesting an abiotic transfer pathway into plant tissue.

## Conclusion

We found evidence for the formation of CENs in the transport of soil resources, evaluated as isotope enrichment. This opens the possibility of ecologically relevant belowground networks beyond CMNs. The interconnection of concurrent networks by different fungal guilds could form an extensive network providing a multitude of functions, which may be unique across ecosystems where each fungal component plays its distinct role. CFN-RNP could play a central role in those network dynamics and may act similar to compartmentalization in cells, whereby a single fungal species would act like an organelle making an unique contribution to overall functioning. If these networks also form in natural ecosystems, this will reveal an unknown role of endophyte diversity in shaping plant-plant interactions. Future research on CMNs will need to account for the fact that endophytes can also transfer resources, from donor side to receiver plants. From our results, we also identify key distinctions between CENs and CMNs. Most notably, CENs should function differently since endophytes harbor distinct saprotrophic and pathogenic capacities^38^. While some mycorrhizal fungi can decompose organic matter, they are not saprotrophs^39^. Endophytes are generally not known to produce specialized mycorrhizal fungal structures, such as arbuscules, Hartig nets, and mantles, which means the interface of resource transfer is also distinct from mycorrhizal fungi. Further, by making use of the well-described genome of *A.thaliana*, future work could aim at disentangling the mechanisms of endophyte-mediated resource transfer and comparing them to pathways found in mycorrhizal symbioses. In conclusion, we propose an unknown role of endophytes in ecosystem functioning which could have important implications for ecosystem management where the current focus concentrates on mycorrhizal fungi.

## Materials and methods

### Experimental design

The experiment was set up in three-way split petri dishes to control for direct plant root connection and to facilitate fungal colonization of donor and receiver plants. The plates were constructed from triple-split petri dishes where two 2 cm holes were melted into the lids to allow plant outgrowth. In contrast to earlier approaches for studying CMNs^5,43,44^, we chose an approach without a mesh barrier. Since all endophytes used in this study overgrow internal barriers of triple-split petri dish plates (Suppl. Figure 2), there was no need for mesh to establish barriers to exclude root contact since roots did not come into contact even without mesh and this approach also better allowed us to control for soil water flow. The plates were sterilized by sequential treatment with 5% sodium hypochlorite (30 min), sterile water rinsing, 70% ethanol (15 min), and UV exposure (30 min) in a laminar flow hood. The holes were sealed with parafilm until fungal inoculation to maintain sterility. For plate assembly, Modified Melin Norkans (MMN) agar was poured into one of the three plate compartments to the level of the internal barriers to support hyphal growth into the two soil compartments. The agar compartments were covered with white paper to simulate soil darkness. The plants were transferred with tweezers into a sterile growth substrate consisting of equal parts sand, vermiculite, and potting soil.

One endophyte species was inoculated per plate and the isotopes were exclusively added to the donor side. The experiment consisted of four separate treatments where E = endophyte, I = isotope, R = receiver plant, and ‘+’ and ‘-’ refer to the presence or absence of an experimental level, respectively: (1) + E/+I/+R, (2) + E/+I/-R, (3) -E/+I/+R, (4) -E/-I/+R. Additionally, there were three endophyte species levels, two isotope addition levels (organic and inorganic nitrogen source), and three measured isotopic elements (^15^N, ^13^C, deuterium). The organic versus inorganic N treatments represent the two opposing ^15^N-labeled nitrogen sources (ammonium versus amino acids; see below).

In the + E/+I/+R treatment, a recipient and a donor plant were inoculated with an endophyte and both isotope treatments (α, ß) (6 replicates per species and per isotope treatment). In the + E/+I/-R treatment, a donor plant was inoculated with an endophyte without a receiver plant and with only one isotope treatment (α) to account for the potential deposit of isotope-containing compounds through fungal exudates and necromass (4 replicates per species). The -E/+I/+R treatment was an isotope control where no endophyte was added but both isotope treatments (α, ß) were included to test for transport mechanisms in the absence of inoculated endophytes (5 replicates per isotope treatment). Finally, the -E/-I/+R treatment served as our natural abundance control without endophytes nor isotopes (10 replicates).

### Choice of plant and endophyte species

The organisms used in this study were *Arabidopsis thaliana* (Col-0), *T. viride*,* F. temperatum*, and *M. hiemalis*. *Arabidopsis thaliana* (Col-0) seeds were obtained from the Eurasian Arabidopsis Stock Centre (uNASC), an established public research repository. The fungal isolates (*Trichoderma viride*,* Fusarium temperatum*,* and Mucor hiemalis*) were obtained from MyPilz for research purposes (https://mypilz.eu/) and are publicly available for research purposes upon request. Species identification of the fungal isolates were provided by the supplier using DNA metabarcoding. No field collection of plant or fungal material was conducted in this study. As all biological materials were acquired from authorized providers and not collected in situ, no collection permits, geographical coordinates, or wild-origin voucher specimens apply. All materials were used in accordance with institutional, national, and international regulations.

*A. thaliana* seeds were first cold stratified at 4 °C for 48 h, sown into a non-sterile potting soil (this was necessary to promote plant growth which was much lower in sterilized potting soil), and after 36 days when plants had an established rosette, plants were then transferred to the plates with inoculated endophytes. The fungal strains were cultivated on MMN agar and hyphae from the growing periphery containing agar plugs (0.5 cm³) were used for inoculation. While germinating *Arabidopsis* in unsterilized potting soil may allow exogenous fungi to colonize its roots, plants were subsequently transferred into a sterile environment in the plates with high propagule pressure of the inoculated fungus.

*F. temperatum* is primarily regarded as a hemibiotrophic pathogen that possesses saprotrophic and parasitic abilities to colonize roots^30–32^. While *Fusarium* often acts pathogenically, many can also behave as mutualistic endophytes^45^, and *F. temperatum* can colonize Brassicaceae root systems^30^. For *F. temperatum*, we used the strain MP-PP9 which was isolated from a maize plant infected with *Ustilago maydis* near Riegersburg, Austria. *Mucor* species can enhance plant growth^46,47^, and *M. hiemalis* can specifically promote host plant nutrient uptake and confer protection against pathogens^48,49^. *M. hiemalis* is considered a saprotrophic fungus capable of endophytically colonizing roots^50,51^ and it can form symbioses with plants similar to mycorrhizae and ‘Mucoromycotina fine root endophytes’ (MFRE)^52–54^. MFRE have been shown to facilitate direct nitrogen, phosphorus and carbon exchange with a host^54^. For *M. hiemalis*, we used the strain MP-304-2-R1 which was isolated from roots in a soil sample near Falkenstein, Austria. Fungi from the genus *Trichoderma* are considered free-living saprotrophs that can be especially beneficial to plants as endophytes by colonizing their roots, and *A. thaliana* has been studied extensively for its responses to *Trichoderma* species^18,35,50,55–59^. *T. viride* can enhance plant growth and confer pathogen protection within *A. thaliana*^35,36,60,61^. For *T. viride*, we used the strain MP-206-8 which was isolated from soil near Gutenstein, Austria. In summary, all three species are capable of colonizing plant roots and are expected to follow a continuum of mutualistic effects whereby *Trichoderma* > *Mucor* > *Fusarium*.

*F. temperatum* was introduced to the plates five days before, and *T. viride* and *M. hiemalis* four days before plant addition to ensure growth dominance of the inoculated fungi over those potentially carried over from the plant roots from the non-sterile soil (see above). *F. temperatum* is fast growing but less quickly than *T. viride* and *M. hiemalis*. For fungal inoculation, the plates were incubated at 28 °C. After plant addition, the plates were incubated in a growth chamber with a constant humidity of 65% for 16 days at 20 °C and 300 µmole/m^2^s light density at day and 15 °C and no light at night (12 h). During the first three days, twelve non-viable plants were replaced with healthy individuals. Watering until moist but not oversaturated with water continued every two days with care taken to avoid cross-contamination between compartments by over-watering. Four plants were removed due to mortality (two from the control and two from inoculation with *M. hiemalis*).

### Isotope additions

Isotopes were added to assess nitrogen, carbon, and water transfer from donor to receiver plant compartments. Four days after plant addition, soil samples from three plates (one per fungal species) were collected for total soil nitrogen analysis to determine how much labelled amino acid to spike into the donor compartment. Total nitrogen was quantified on finely ground soils using dry combustion on an Elemental Analyzer coupled to an isotope ratio mass spectrometer (EA-IRMS), which was also used to quantify isotope enrichment (see below). This was used to estimate total amino acid content in the soil assuming amino acids are ca. 33% of the total N content^62–66^. We also measured ammonium content using a modified Berthelot reaction ^67,68^ from 2 M KCl extractions (5 g soil, 20 mL KCl). We then added labelled substrates corresponding in amount to only 1% of the already present soil pools to avoid a fertilization effect. Isotopic treatments α (^13^C-amino acid mixture and ^15^NH₄) and β (^15^N-amino acid mixture) were applied to the donor plants after 16 days of plant growth.

We used highly enriched substrates, including ammonium-^15^N_2_ sulfate (99 atom % ^15^N) and 98% ^13^C/^15^N atom content mixed amino acids extracted from algae (Cambridge Isotope Laboratories ALGAL AMINO ACID MIXTURE). To minimize exchange of deuterium in the water, the deuterium (D) treatment was applied for both isotope treatments only 48 h before sampling (after 22 days) by adding 400 µl 20% D_2_O (99 atom % D). We used isotopically labelled amino acids, ammonium, and water to test different transfer outcomes among CFNs. We added both ^13^C- and ^15^N-labelled amino acids to assess whether endophytes may also transfer carbon from amino acids (versus just N), and we added ^15^N-NH₄ to assess transport of nitrogen from an inorganic versus organic source.

### Sampling and analysis

Sampling was conducted 24 days after plant transfer to inoculation plates. Plants were removed from the plates and separated into above and belowground parts. Leaves and shoots (hereafter: “plant”; we only used above-ground plant parts as we cannot differentiate isotope uptake into plant versus fungal tissues in and around the roots) and soils were dried at 60 °C for 72 h and ground for analysis on an elemental analyzer coupled to an isotope-ratio mass spectrometer (EA-IRMS) to determine isotopic incorporation into aboveground tissues and transfer into soils. The roots were stained with trypan blue to assess fungal colonization using the grid-intersection method (McGONIGLE et al., 1990). A light microscope was used to evaluate colonization at 40x magnification by dividing roots into 50 sections and scoring the presence or absence of hyphae.

### Statistical analysis

All statistical analyses were conducted in R (v4.3.1), and significance was set to *P* ≤ 0.05. ANOVAs (aov function), F-tests (var.test function), t-tests (t.test function), and Pearson correlations (cor.test function) from base R were used for the analyses. For the isotope addition treatments, we first tested whether the isotope control and natural abundance control treatments differed to evaluate potential non-fungal mediated resource transfer, evaluated as isotope enrichment. We tested whether variance was equal between the samples using F-tests and then used two-sided Welch’s t-tests if the variance was unequal (setting var.equal = FALSE) and two-sided Student’s t-tests if the variance was equal. If the isotope and natural abundance controls differed, then we then tested for differences among fungal treatments against both controls. If the controls did not differ, we compared fungal differences to the natural abundance control. To test this, we used ANOVA and calculated type III sums of squares. Normality of the residuals was inspected, and when necessary, the data was log transformed. Kruskal-Wallis rank sum tests (kruskal.test function) were used as a non-parametric alternative when the log-transformed model residuals were not normally distributed or when a log transformation was not possible due to negative values, such as observed for foliar δ^13^C. To test whether individual fungal treatments increased isotope values relative to the control and for the physiological analyses, we first tested whether the variance of samples was equal using F-tests, and we then used one-sided Welch’s t-tests for samples with unequal variance and one-sided Student’s t-tests for samples with equal variances.

## Supplementary Information

Below is the link to the electronic supplementary material.

Supplementary Material 1

## Data Availability

All analysis scripts and data are accessible in the following repository: [https://gitlab.com/fungalecology/cen].
