# Supplementary material for: Evidence for resource transfer via common endophyte networks
Source: Sci Rep. 2026 Jun 8;16:26111. doi: 10.1038/s41598-026-56653-9 (PMC13490404; doi:10.1038/s41598-026-56653-9)
Supplement: Supplementary file 1 [file 41598_2026_56653_MOESM1_ESM.pdf]

Supplementary Materials

Evidence for resource transfer via common endophyte networks

Philipp Waschk[1,*], Philipp Spiegel[1], Arthur Gessler[2,3], Matthias Saurer[2], Beatrice M. Bock[4,5], Wolfgang Hinterdobler[6], Mark A. Anthony[1,*]

[1] Center for Microbiology and Environmental Systems Science, University of Vienna, Vienna, Austria.
[2] Swiss Federal Institute for Forest, Snow, and Landscape Research (WSL), Birmensdorf, Switzerland
[3] Department of Environmental Systems Science, ETH Zürich, Zürich, Switzerland.
[4] Department of Biological Sciences, Northern Arizona University, Flagstaff, AZ, USA
[5] Center for Adaptable Western Landscapes, Northern Arizona University, Flagstaff, AZ, USA
[6] MyPilz GmbH, Vienna, Austria
[*] Correspondence should be addressed to these authors: Mark A. Anthony (mark.anthony@univie.ac.at), Philipp Waschk (pwaschk.research@gmail.com)

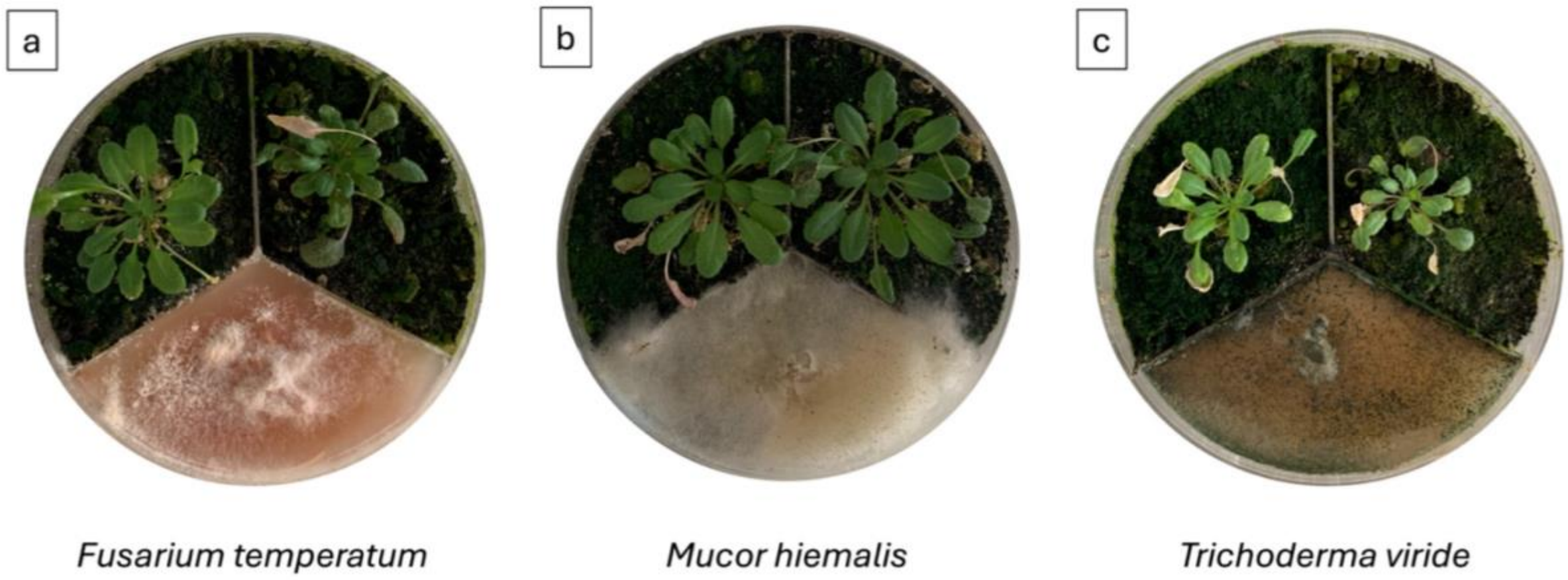

Supplement Figure 1: Pictures of the inoculated plates. Images were taken right before sampling. Inoculation with *F. temperatum* (**a**), *Mucor hiemalis* (**b**) and *T. viride* (**c**).

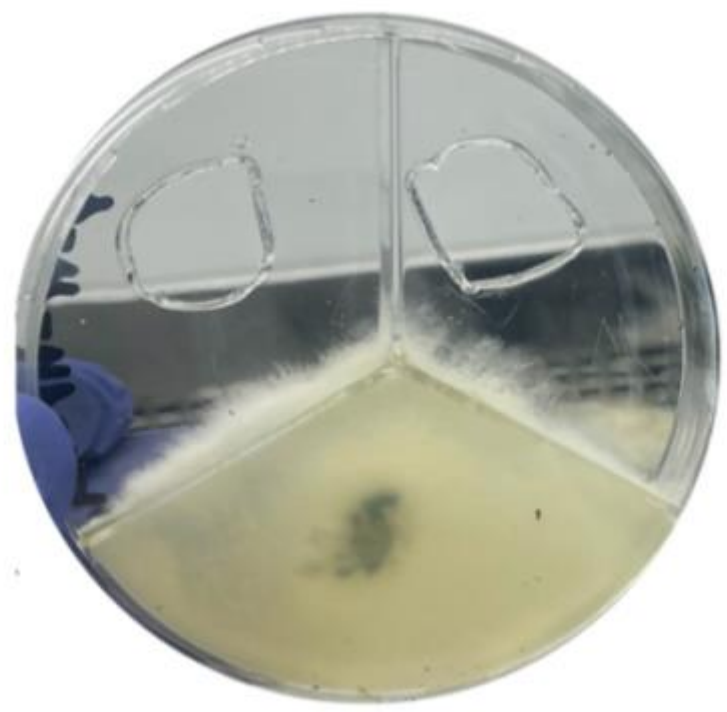

Supplement Figure 2: Pictures of the overgrowth of the internal barriers by *Mucor hiemalis*.

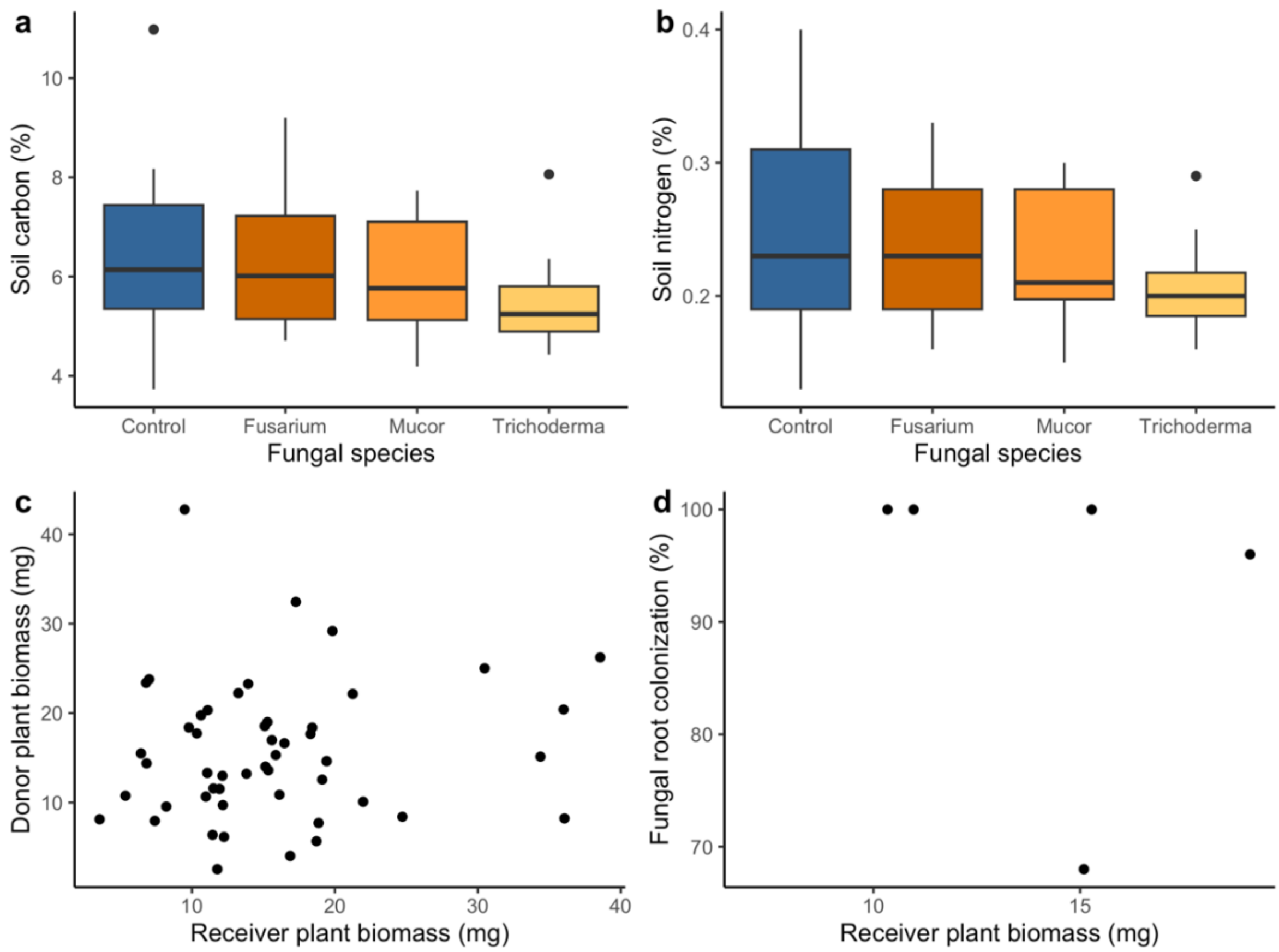

Supplement Figure 3: Validation of the experimental system. Fertility of the receiver soils as total carbon and nitrogen (**a**, **b**). Correlation of the biomasses of the donor and receiver plant ($p = 0.43$, $R = 0.11$)(**c**). Correlation of fungal root colonization and plant biomass of only control treatments ($p = 0.7$, $R = -0.25$)(**d**).

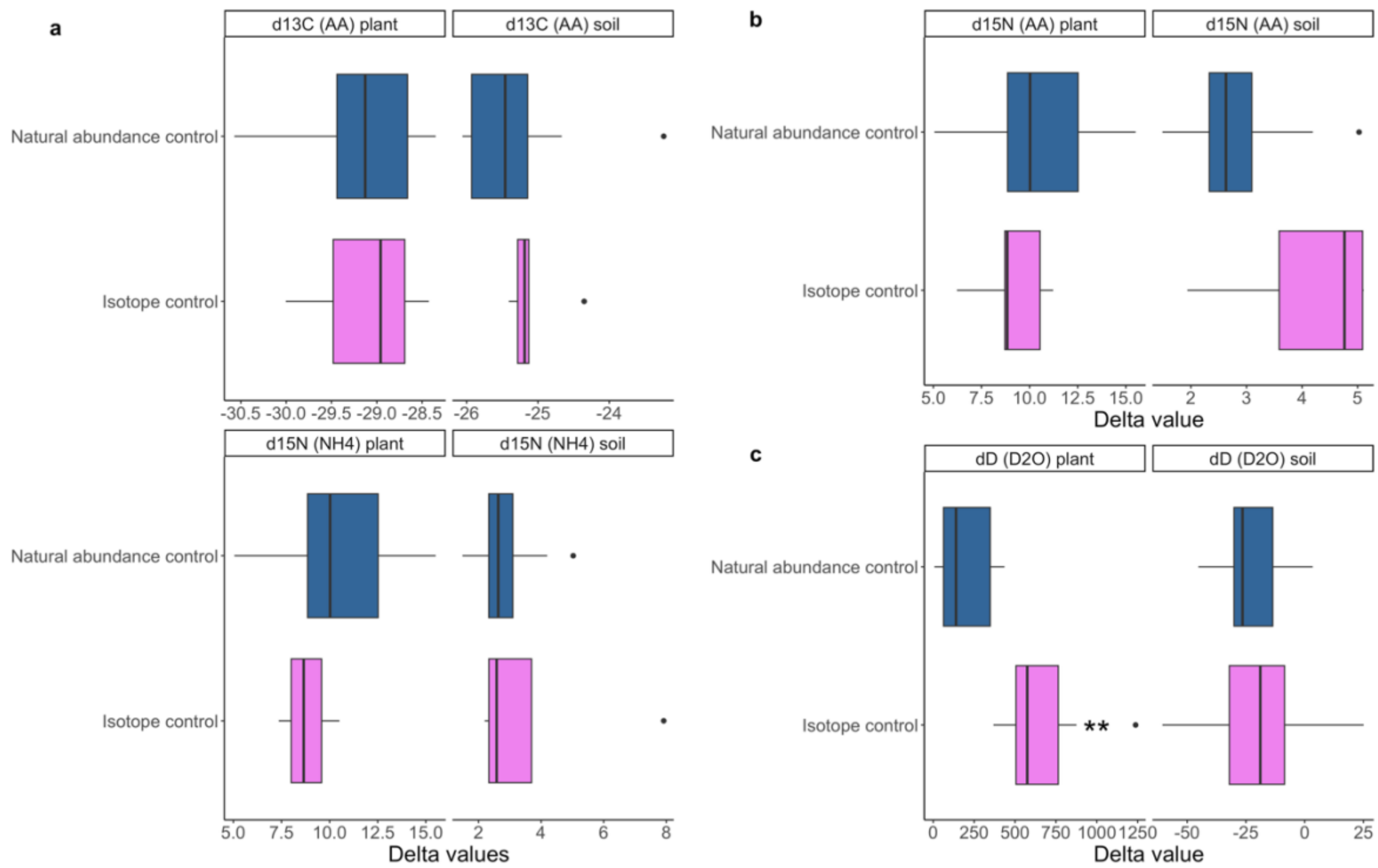

Supplement Figure 4: Comparison of isotope and natural abundance controls. Comparison of isotope and natural abundance controls of the control plates where ^{13}C was added as amino acids and ^{15}N as ammonium (**a**). Comparison of isotope and natural abundance controls of the control plates where ^{15}N was added as amino acids (**b**). Comparison of isotope and natural abundance controls of the control plates where deuterium was added as D_2O showing significantly more δD in the isotope control compared to the natural abundance control ($p = 0.0007$) (**c**). (** means $p \leq 0.01$).

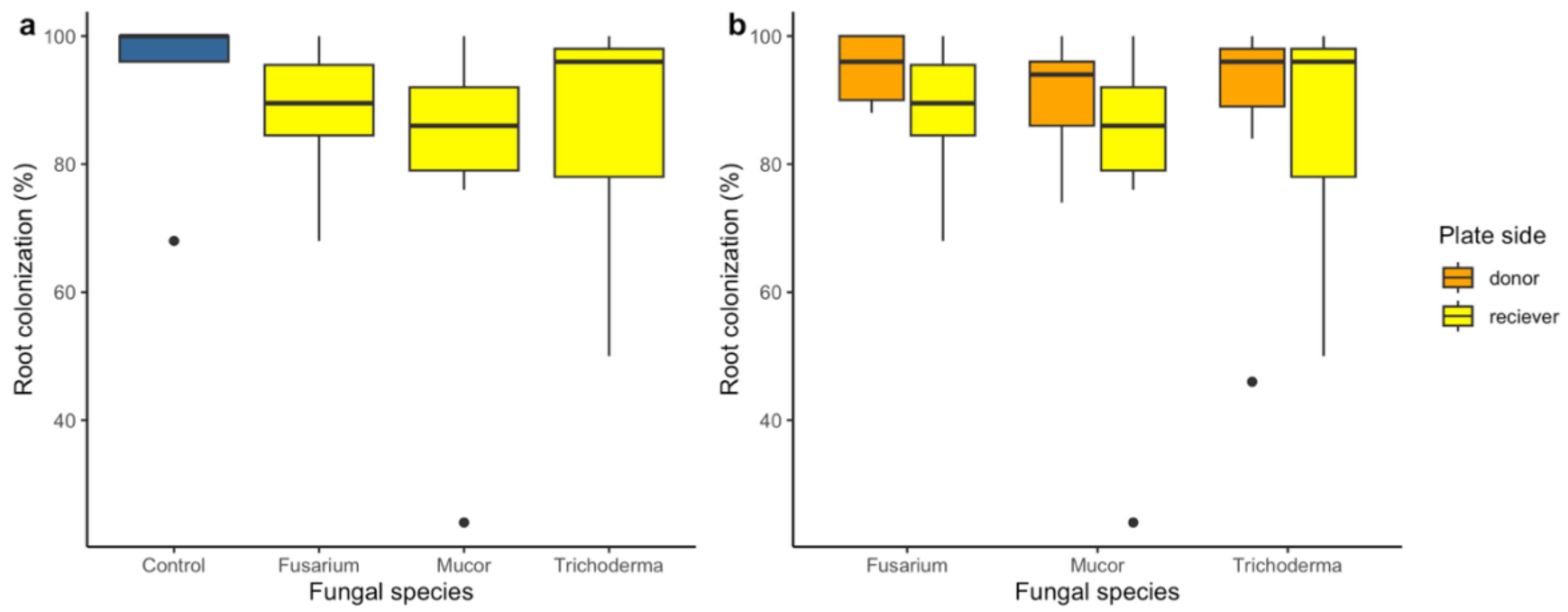

Supplement Figure 5: Fungal root colonization. Comparison of fungal root colonization of receiver plants to the natural abundance control by endophyte species (**a**). Comparison of fungal root colonization of receiver and donor plants by endophyte species (**b**).

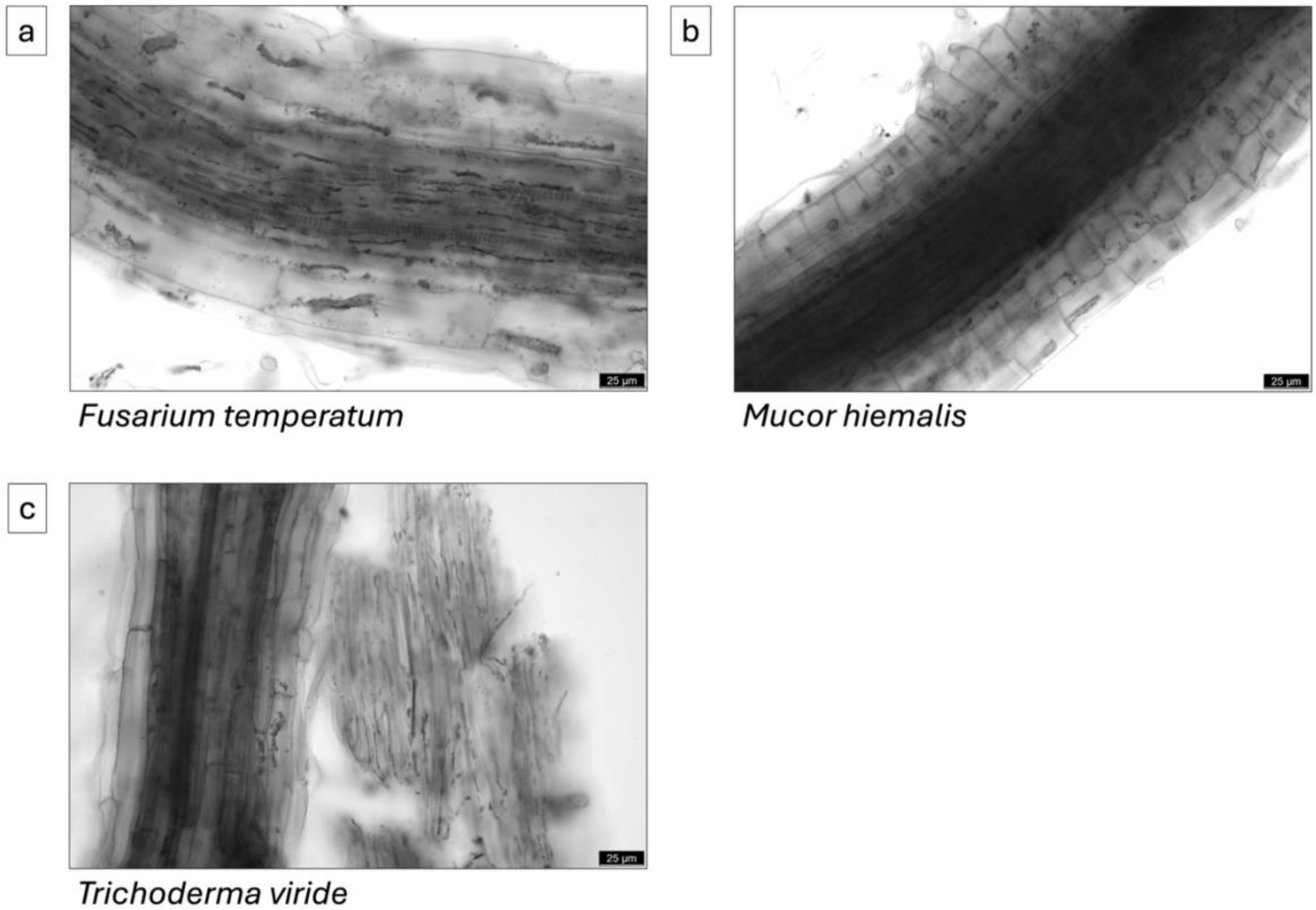

Supplement Figure 6: Light microscope images showing fungal colonization of trypan blue stained root tissues of inoculations of *A. thaliana* with *Fusarium temperatum* (**a**), *Mucor hiemalis* (**b**), and *Trichoderma viride* (**c**).

Supplement Table 1: Statistical values (Correlations, t-tests). Table of correlations and t-tests showing comparison references and corresponding p-values and R-values (blue means p ≤ 0.05; violet means p ≤ 0.01).

Statistical method	Comparison reference 1	Comparison reference 2	p	R
Pearson correlation	Donor plant	Receiver plant	0.4326	0.1134861
Pearson correlation	Fungal root colonization of reciever plants (only control)	Plant biomass	0.6878	-0.2477715
Pearson correlation	Fungal root colonization of reciever plants (only fungal inoculations)	Plant biomass	0.01106	0.5091308
One-sided Welch's t-test	Plant biomass (*Fusarium temperatum*)	Plant biomass (Natural abundance control)	0.01387	
One-sided Welch's t-test	Plant biomass (*Trichoderma viride*)	Plant biomass (Natural abundance control)	0.07448	
One-sided Student's t-test	Total nitrogen content (*Mucor hiemalis*) in plants (plates with receiver plants)	Total nitrogen content (Natural abundance control) in plants (plates with receiver plants)	0.001003	
Two-sided Student's t-test	∂D(D_2O) (Natural abundance control) in plants (plates with receiver plants)	∂D(D_2O) (isotope control) in plants (plates with receiver plants)	0.0006808	
One-sided Student's t-test	∂^{13}C(AA) (*Mucor hiemalis*) in soils (plates without receiver plants)	∂^{13}C(AA) (Natural abundance control) in soils (plates with receiver plants)	0.05391	
One-sided Welch's t-test	∂D(D_2O) (*Trichoderma viride*) in plants (plates without receiver plants)	∂D(D_2O) (Natural abundance control) in plants (plates with receiver plants)	0.04365	
One-sided Student's t-test	∂^{13}C(AA) (*Trichoderma viride*) in plants (plates with receiver plants)	∂^{13}C(AA) (Natural abundance control) in plants (plates with receiver plants)	0.04962	
One-sided Student's t-test	∂^{15}N(AA) (*Fusarium temperatum*) in plants (plates with receiver plants)	∂^{15}N(AA) (Natural abundance control) in plants (plates with receiver plants)	0.01683	
One-sided Student's t-test	∂^{15}N(AA) (*Fusarium temperatum*) in soils (plates with receiver plants)	∂^{15}N(AA) (Natural abundance control) in soils (plates with receiver plants)	0.001306	
One-sided Student's t-test	∂^{15}N(AA) (*Mucor hiemalis*) in soils (plates with receiver plants)	∂^{15}N(AA) (Natural abundance control) in soils (plates with receiver plants)	0.006907	
One-sided Student's t-test	∂D(D_2O) (*Fusarium temperatum*) in soils (plates with receiver plants)	∂D(D_2O) (Natural abundance control) in soils (plates with receiver plants)	0.00001287	
One-sided Welch's t-test	∂D(D_2O) (*Mucor hiemalis*) in soils (plates with receiver plants)	∂D(D_2O) (Natural abundance control) in soils (plates with receiver plants)	0.04282	
One-sided Welch's t-test	∂D(D_2O) (*Fusarium temperatum*) in plants (plates with receiver plants)	∂D(D_2O) (Natural abundance control) in plants (plates with receiver plants)	0.002062	
One-sided Welch's t-test	∂D(D_2O) (*Mucor hiemalis*) in plants (plates with receiver plants)	∂D(D_2O) (Natural abundance control) in plants (plates with receiver plants)	0.001896	
One-sided Welch's t-test	∂D(D_2O) (*Trichoderma viride*) in plants (plates with receiver plants)	∂D(D_2O) (Natural abundance control) in plants (plates with receiver plants)	0.01153	

Supplement Table 2: Statistical values (ANOVAs). Table of ANOVAs and Kruskal-Wallis rank sum tests showing the data that has been tested for fungal species effects compared to the natural abundance control and corresponding p-values (blue means p ≤ 0.05; violet means p ≤ 0.01).

Statistical method	Data	Comparison	p
ANOVA	∂D in soils (plates without receiver plants)	Fungal species	0.0266
Kruskal-Wallis rank sum test	∂D in soils (plates without receiver plants)	Fungal species	0.0485
ANOVA	Total plant nitrogen	Fungal species	0.001038
ANOVA	$\partial^{13}C$(AA) in plants (plates with receiver plants)	Fungal species	0.05292
ANOVA	$\partial^{15}N$(AA) in soils (plates with receiver plants)	Fungal species	0.01
ANOVA	∂D(D_2O) in plants (plates with receiver plants)	Fungal species	0.02039
ANOVA	∂D(D_2O) in soils (plates with receiver plants)	Fungal species	0.0004977
Kruskal-Wallis rank sum test	∂D(D_2O) in soils (plates with receiver plants)	Fungal species	0.0001153
